# Pre-treatment alphafeto protein in hepatocellular carcinoma with non-viral aetiology – a prospective study

**DOI:** 10.1186/s12876-017-0710-x

**Published:** 2017-12-06

**Authors:** Siriwardana Rohan Chaminda, Thilakarathne Suchintha, Niriella Madunil Anuk, Dassanayake Anuradha Supun, Gunathilake Mahen Bhagya, Liyanage Chandika Anuruddha Habarakada, De Silva Hithadurage Janaka

**Affiliations:** 1 Department of surgery, Faculty of Medicine, University of Kelaniya Sri Lanka, Kelaniya, Sri Lanka; 2 Department of medicine, Faculty of Medicine, University of Kelaniya Sri Lanka, Kelaniya, Sri Lanka; 3 Department of pharmacology, Faculty of Medicine, University of Kelaniya Sri Lanka, Kelaniya, Sri Lanka

**Keywords:** Carcinoma hepatocellular/diagnosis, Carcinoma hepatocellular/aetiology, Alpha-fetoproteins/analysis, Prognosis

## Abstract

**Background:**

Alpha-fetoprotein (AFP) is a biomarker for hepatocellular carcinoma (HCC). The significance of pre-treatment AFP (pt-AFP) in non-viral HCC (nvHCC) is not clear.

**Methods:**

Patients with nvHCC, referred to a Hepatobiliary Clinic from September 2011–2015 were screened. HCC was diagnosed using American Association for the Study of Liver Disease guidelines, and TNM staged. nvHCC was diagnosed when HBsAg and anti-HCVAb was negative. Child-Turcotte-Pugh (CTP) and Model for End-stage Liver Disease (MELD) scores were calculated. AFP level was evaluated against patient characteristics, tumour characteristics and survival.

**Results:**

Three hundred eighty-nine patients with nvHCC [age 64(12–88) years; 344(88.4%) males] were screened. Median AFP was 25.46 ng/ml (1.16–100,000). 41.2% (*n* = 160) Of patients had normal AFP level. 22.9% (*n* = 89) had AFP over 400 ng/ml. Female gender (*P* < 0.05), vascular invasion (*P* < 0.001), tumours over 5 cm (*P* < 0.05), late TNM stage (*P* < 0.001) and non-surgical candidates had higher AFP levels. Diffuse type (*P* < 0.001), macro vascular invasion (*P* < 0.001) and late stage tumours (*P* < 0.001) had AFP over 400 ng/ml. Having AFP below 400 ng/ml was associated with longer survival (16 vs. 7 months, *P* < 0.001).

**Conclusion:**

Pre treatment AFP has a limited value In diagnosing nvHCC, Having a AF*P* value over 400 ng/ml was associated with aggressive tumour behaviour and poor prognosis.

## Background

Hepatocellular carcinoma (HCC) is the second leading cause of cancer death worldwide [1]. Half of all cases of HCC are associated with hepatitis B virus infection and 25% associate with hepatitis C virus [2]. In absence of viral aetiology other factors such as alcoholic liver disease, non - alcoholic steato - hepatitis (NASH), intake of aflatoxin contaminated food, diabetes, and obesity [3, 4] are secondary risk factors. In Sri Lanka, great majority of patients with HCC are not associated with viral aetiology [5] in keeping with the low prevalence of Hepatitis B and C [6, 7] in the country. Non-alcoholic fatty liver disease (NAFLD) and non-alcoholic steato-hepatitis (NASH) are becoming increasingly prevalent in the Asian continent and are already a leading cause of chronic liver disease in the region [8–10].

Serum alpha-fetoprotein (AFP) is a commonly used screening biomarker in patients at risk for HCC. Use of AFP in diagnosing HCC has been debated due to its variability in sensitivity and specificity [11]. It has been recognized as a poor prognostic factor in patients with HCC. Hence serum AFP level is included in some HCC staging systems [12]. It has also found to vary with the etiology of liver disease [13]. There is paucity of data on the significance of pre treatment alpha–fetoprotein in HCC patients of non-viral aetiology. This study analyse the significance of pre treatment alpha-fetoprotein in a cohort of patients with non-viral HCC.

## Methods

Three hundred and ninety patients with hepatocellular carcinoma (HCC) were referred from all around the country from 2011 September to 2015 September. One patient was infected with both hepatitis B and C viruses and excluded from the study. Rest of the patients (*n* = 389) with hepatocellular carcinoma of non-viral aetiology were selected for the study. Work up of patients included detailed history and examination, haematological investigations including pre-treatment serum alpha - feto protein (AFP) level and imaging with contrast enhanced CT scans of the abdomen. HCC was diagnosed according to the American Association for the Study of Liver Diseases (AASLD) guidelines [14].

Biopsy from the lesion was done only in 4 patients with atypical imaging. A detailed history was taken to assess the degree of alcohol consumption. Patients who had a history of consuming alcohol above the accepted safe limits (Asian standards: <14 units of alcohol per week in men and <7 units per week in women) prior to the diagnosis of cirrhosis were considered as having alcoholic cirrhosis. Patients who did not drink alcohol above the safe limit, had no history of contributing drug or herbal product, who were hepatitis B surface antigen and C antibody was negative, absence of auto immune disease, normal serum ferritin levels and having a normal serum copper levels were taken as cryptogenic cirrhotics.

Staging of the cancer was done according to TNM classification developed jointly by the American Joint Committee on Cancer (AJCC) and the International Union for Cancer Control (UICC). Child-Turcotte-Pugh (CTP) and Model for end stage liver disease (MELD) scores were calculated for further prognostication. Non-viral aetiology of the HCC was confirmed by Hepatitis B surface antigen testing and Hepatitis C antibody testing.

Decisions regarding liver transplantation, surgical resection, ablation, trans-arterial chemoembolization or Sorafenib therapy were made according to tumor morphology, background liver status and functional index. Management decisions were taken at a multi-disciplinary meeting. A team of dedicated hepato biliary surgeons performed surgery. The patients were followed up in a special combined medical and surgical clinic at three monthly intervals. CECT abdomen was performed three monthly in the first year and six monthly in the next two years. All the data were entered in to a database prospectively.

Data are presented as mean with standard deviation (SD), median with interquartile range (IQR) and frequencies with percentages (%). The differences between groups were evaluated using Pearson’s Chi-square test, Mann Whitney U, Kruskal-Wallis Test as appropriate. Initially single variable analysis was done to screen the variables and subsequently multiple variable analysis were carried out to determine association between variables. Cumulative survival and recurrence rates were calculated using the Kaplan - Meier method and the difference between survivals was evaluated using the log - rank test. A *P* value of less than 0.05 was considered statistically significant. IBM SPSS Statistics V22.0 was used for statistical analysis.

## Results

The median age of the study population was 64 (range 12–88) years and 88.5% were males. Sixty per cent were diagnosed patients with diabetes mellitus. 48% Were regular alcohol consumers while 20.4% were social drinkers and 31.6% were non-alcohol consumers. Background cirrhosis was noted in 84.5% of the patients. The Child - Pugh class A, B and C had 57.3%, 32.4% and 10.3% patients respectively. The median Child - Pugh score was 6 (range1–14). The median MELD score was 11 (range 5–28). Majority (45.5%) had stage III HCC. The median total tumour diameter was 6 cm (0.9–26.5). Sixty three per cent of the patients had no signs of vascular or visceral invasion. The median INR was 1.23 (0.97–98).

**Fig. 1:**
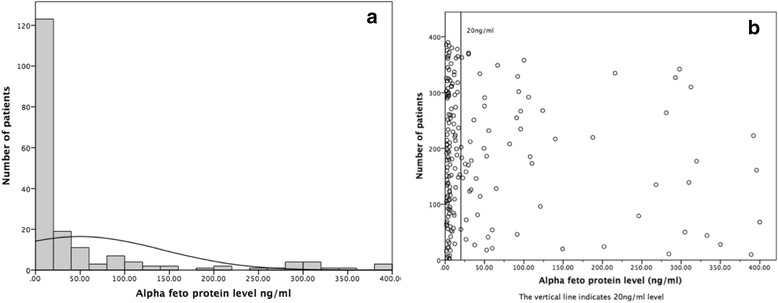
The distribution of alpha feto-protein level (graph truncated at AFP 400 ng/ml). **a** Histogram, **b** Scatter plot

The median alpha feto protein level was 25.46 ng/ml (range 1.16–100,000). Only 41.2% patients had an AFP level above the reference range (0–10 ng/ml) (Fig. 1). Twenty three percent of the patients had AFP level above 400 ng/ml.

The median AFP level was compared in different sub groups (Table 1). In regular alcohol consumers, median AFP level was 38.5 ng/ml while in others median AFP was 18.57 ng/ml showing no statistical difference (*P* = 0.197). Female gender was associated with statistically significant higher median AFP level (*P* = 0.049). Tumours with vascular invasion had a higher median AFP value (*P* = 0.002). HCC with total tumour diameter more than 5 cm (*P* = 0.013) and diffuse type tumours (*P* = 0.002) had higher AFP levels. Late tumour stage (stage 3 and 4) was associated with a higher median AFP value compared to early stage tumours (*P* = 0.001).

**Table 1:** Comparison of median AFP among groups of patient

Factor (number within brackets)	AFP level (ng/ml) median and range		*P* value
Gender (Males 343, 88.2%)	Male 20 (1.16–100,000)	Female 268 (3–26,172)	0.049
Macro vascular invasion (Present 110, 28.3%)	Present 908 (1.16–100,000)	Absent 12.6 (1.48–92,120)	0.002
Tumour diameter (<5 cm 176, 45.2%)	<5 cm 11.5 (1.84–50,000)	>5 cm 32 (1.16–94,120)	0.013
Stage (Early 196, 50.4%)	Early 10.54 (1.48–50,000) (Stage I/II)	Late 202 (1.16–100,000) (Sage III/IV)	0.001
Macroscopic tumour (Nodular 269, 69.1%)	Nodular 9.6 (1.16–50,000)	Diffuse 2308.5 (1.84–100,000)	0.002
Cirrhotic state (Cirrhotics 301, 77.4%)	Cirrhotic 20.4 (1.48–10,000)	Non cirrhotic 65 (1.16–94,120)	0.150
Alcohol consumption (Alcoholics −266, 68.4%)	Alcoholic 26.6 (1.48–94,120)	Non alcoholic 21 (1.16–100,000)	0.197
Diabetes mellitus (Diabetics 234, 60.1%)	Diabetics 26.6 (1.48–100,000)	Non diabetics 22 (1.16–94,120)	0.808
Child-Pugh (Class A - 223, 57.3%)	Class A 12.33(1.66–94,120)	Class B/C 26.03 (1.76–100,000)	0.179
Tumour nodularity (Single 199, 51.2%)	Single 9.6 (1.16–50,000)	Multiple 17.36 (1.85–94,120)	0.119

21% (*n* = 81) of patients underwent curative resection, three patients were offered liver transplantation. Radiofrequancy and alcohol ablation was performed in 10% (*n* = 38). Trans arterial chemo embolization was done in 34% (*n* = 131). 35% (*n* = 136) of the patients were offered Sorafinib or palliative care. Patients who were candidates for surgical treatment had lower median AFP level compared to the patients who underwent nonsurgical treatment (*P* = 0.001). Factors such as cirrhosis, diagnosis of diabetes mellitus, Childs class, state of non-tumour liver, bile duct invasion, recurrence of tumour after treatment, metastatic tumour at presentation and number of tumour nodules were not associated with significantly elevated median AFP values.

Patients who had AFP less than 400 ng/ml and more than 400 ng/ml were compared in different sub groups. Diffuse tumours (*P* = 0.001), invasive tumours (*P* = 0.002) and late stage tumours (*P* = 0.004) had statistically significant association of having an AFP level above 400 ng/ml. However multi nodular tumours (*P* = 0.992), large tumour diameter (*P* = 0.155) and presence of background cirrhosis (*P* = 0.694), history of alcohol consumption did not have a significant association of having an AFP level above 400 ng/ml.

**Fig. 2:**
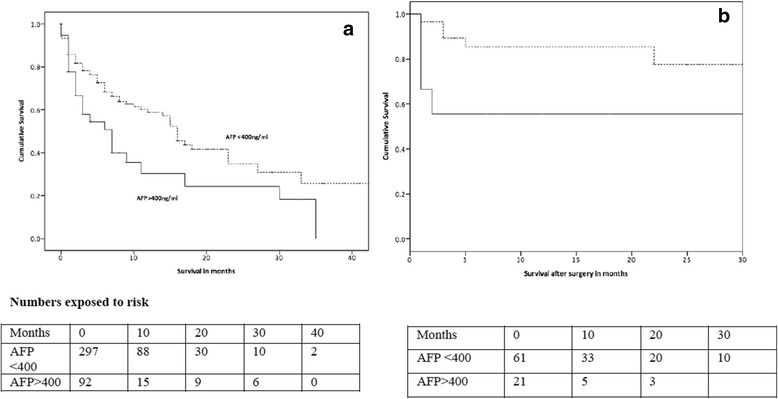
Survival of patients with regard to AFP level. **a** Over all survival of patient who had AFP above and below 400ng/ml(*P*= 0.02), **b** Survival of Patients who underwent surgery- AFP above and below 400ng/ml(*P*=0.01)

Patient survival was compared taking AFP of 400 ng/ml level as the cut off point. Patients with AFP value less than 400 ng/ml had a median overall survival of sixteen months and AFP more than 400 ng/ml had only a median survival of seven months (Fig. 2a). There was a statistically significant increased survival in the patient group with an AFP value less than 400 ng/ml (*P* = 0.002). On multivariate Cox regression analysis presence of macro vascular invasion (*P* = 0.940), Child Turcotte-Pugh class (*P* = 0.171), tumour size below or above 5 cm (*P* = 0.068) were not individual predictors. Tumour nodularity (*P* = 0.026), AFP level (*P* = 0.008), and early stage (*P* = 0.042) were individual predictors of survival.

When patients who underwent surgery as the primary treatment modality (Fig. 2b) were analysed, patients who had AFP values less than 400 ng/ml had statistically significant better survival compared to those who had AFP more than 400 ng/ml (*P* = 0.013). How ever there was no difference in survival between two AFP categories among the patients who underwent trans arterial chemo embolization as the primary treatment modality (*P* = 0.545) and the patients who did not undergo any form of treatment (*P* = 0.263).

## Discussion

In this cohort of patients with non-viral aetiology, 41% patients had AFP above the reference range while 23% had AFP over 400 ng/ml. higher percentage of larger, vascular invading and advanced stage tumours had AFP level over 400 ng/ml. Surgical candidates had a lower AFP levels. Having AFP over 400 ng/ml predicted poor outcome after surgery.

Worldwide pattern of aetiology of HCC seem to be changing. Previously dominated hepatitis B and C are falling in incidence due to vaccination and effective treatment [15]. Non-viral HCC is becoming the predominant variety. With this change in pattern of disease, it is worth re-evaluating the previously used criteria in managing HCC.

Serum alpha-fetoprotein is a traditional tumour maker used in HCC screening and surveillance. Sensitivity and specificity of AFP in diagnosing HCC varies widely and depends on the cut off level [16, 17]. Variations in the AFP levels depending on the etiology have been observed before. In a cohort of patients with hepatitis B related HCC, AFP at 200 ng/ml and 400 ng/ml showed a sensitivity of 79.8% and 91.5% respectively. In another study specificity was 50% in HBV-positive patients compared to 78% in HBV-negative patients [18].

Limited number of researchers has looked in to the value of AFP in HCC of non-viral aetiology. In our study population of non-viral aetiology, 58.8% had normal AFP levels (<10 ng/ml). Low levels of AFP positivity was also seen in a study done by Murugavel et al. where they studies a group of HCC patients with mixed aetiology [13]. In their study AFP positivity was 20.5% (negative in 79.5%) when the patients were of non viral aetiology. This is comparable with our findings. Patients with non-viral aetiology seem to have a lower AFP levels. Thomson et al. in a systematic review on screening a cohort of mixed aetiology HCC came to the conclusion that screening ultrasound is cost effective when it is offered to patients with an AFP value more than 20 ng/ml [19]. With This criterion applied to our study it would miss 48% of the patients with HCC. Using AFP as a screening or diagnostic investigation seem to be unreliable in them. Block et al. in their study of molecular pathology of viral HCC indicated high hepatocyte turnover in these patients [2]. Relatively lower AFP in non-viral HCC could be related to low rate of hepatocyte turn over.

In this analysis, having a higher AFP level (more than 400 ng/ml) was associated with a significant reduction in overall survival. This was also apparent when survival was analysed in subgroup of patients who underwent surgery. A similar impact of AFP on survival was noted in patients with HCC having viral aetiology [20]. Similarly, AFP is a well-known marker that predicts the outcome after liver transplantation [15] for HCC.

## Conclusion

AFP level at presentation is a poor indicator for screening and diagnosing HCC in patients with non-viral aetiology. Like in other types of HCC, markedly elevated AFP levels seem to predict prognosis in these patients as well.

## Abbreviations

AASLD: American Association for the Study of Liver Diseases
AFP: alpha-fetoprotein
AJCC: American Joint Committee on Cancer
CECT: contrast enhanced computed tomogram
CTP: Child-Turcotte-Pugh
HBsAg: hepatitis B surface antigen
HCC: hepato cellular carcinoma
HCVAb: hepatitis C viral antibody
INR: international normalised ratio
MELD: model for end stage liver disease
NAFLD: non alcoholic fatty liver disease
NASH: non - alcoholic steato - hepatitis
nv- HCC: non vital hepato cellular carcinoma
pt.-AFP: pre treatment alpha feto protein
SD: standard deviation
TNM: tumour node metastasis
UICC: international Union for Cancer Control

## References

[1] Ferlay J, Soerjomataram I, Dikshit R, Eser S, Mathers C, Rebelo M (2015). Cancer incidence and mortality worldwide: sources, methods and major patterns in GLOBOCAN 2012. Int J Cancer.

[2] Block TM, Mehta AS, Fimmel CJ, Jordan R (2003). Molecular viral oncology of hepatocellular carcinoma. Oncogene.

[3] Sanyal AJ, Yoon SK, Lencioni R (2010). The etiology of hepatocellular carcinoma and consequences for treatment. Oncologist.

[4] Blum HE, Moradpour D (2002). Viral pathogenesis of hepatocellular carcinoma. J Gastroenterol Hepatol.

[5] Siriwardana RCLCAH, Gunethileke MB. Hepatocellular carcinoma in Sri Lanka - where do we stand? The Sri Lanka journal of. Surgery. 2013;31(2)

[6] Senevirathna D, Amuduwage S, Weerasingam S, Jayasinghe S, Fernandopulle N, Hepatitis C (2011). Virus in healthy blood donors in Sri Lanka. Asian J Transfus Sci.

[7] Vitarana T. Viral hepatitis in Sri Lanka. Ceylon Med J 1989;34(4):163–177. 2627726

[8] Siriwardana RN, Niriella MA, Liyanage CA, Wijesuriya SR, Gunathilaka B, Dassanayake AS (2013). Cryptogenic cirrhosis is the leading cause for listing for liver transplantation in Sri Lanka. Indian J Gastroenterol.

[9] Dassanayake AS, AK S, Rajindrajith U, Kalubowila S, Chakrawarthi AP (2009). De Silva. Prevalence and risk factors for non-alcoholic fatty liver disease among adults in an urban Sri Lankan population. J Gastroenterol Hepatol.

[10] Chitturi SFG, George J (2004). Non-alcoholic steatohepatitis in the Asia-Paci c region: future shock?. J Gastroenterol Hepatol.

[11] Lok AS, Sterling RK, Everhart JE, Wright EC, Hoefs JC, Di Bisceglie AM (2010). Des-gamma-carboxy prothrombin and alpha-fetoprotein as biomarkers for the early detection of hepatocellular carcinoma. Gastroenterology.

[12] Subramaniam S, Kelley RK, Venook APA (2013). Review of hepatocellular carcinoma (HCC) staging systems. Chin Clin Oncol.

[13] Murugavel KG, Mathews S, Jayanthi V, Shankar EM, Hari R, Surendran R (2008). Alpha-fetoprotein as a tumor marker in hepatocellular carcinoma: investigations in south Indian subjects with hepatotropic virus and aflatoxin etiologies. Int J Infect Dis.

[14] Bruix J, Sherman M (2011). American Association for the Study of liver D. Management of hepatocellular carcinoma: an update. Hepatology.

[15] Kaplan DE, Reddy KR (2003). Rising incidence of hepatocellular carcinoma: the role of hepatitis B and C; the impact on transplantation and outcomes. Clin Liver Dis.

[16] Sherman M, Peltekian KM, Lee C (1995). Screening for hepatocellular carcinoma in chronic carriers of hepatitis B virus: incidence and prevalence of hepatocellular carcinoma in a north American urban population. Hepatology.

[17] Oka H, Tamori A, Kuroki T, Kobayashi K, Yamamoto S (1994). Prospective study of alpha-fetoprotein in cirrhotic patients monitored for development of hepatocellular carcinoma. Hepatology.

[18] Lee HS, Chung YH, Kim CY (1991). Specificities of serum alpha-fetoprotein in HBsAg+ and HBsAg- patients in the diagnosis of hepatocellular carcinoma. Hepatology.

[19] Thompson Coon J, Rogers G, Hewson P, Wright D, Anderson R, Cramp M (2007). Surveillance of cirrhosis for hepatocellular carcinoma: systematic review and economic analysis. Health Technol Assess.

[20] Huo TI, Huang YH, Lui WY, JC W, Lee PC, Chang FY (2004). Selective prognostic impact of serum alpha-fetoprotein level in patients with hepatocellular carcinoma: analysis of 543 patients in a single center. Oncol Rep.

